# Chronic pain in narcolepsy reflects interacting neurobiological and psychological mechanisms: a mini-review

**DOI:** 10.3389/frsle.2026.1930803

**Published:** 2026-09-25

**Authors:** Maria Munch-Menzer, Birgitte Rahbek Kornum

**Affiliations:** Department of Neuroscience, University of Copenhagen, Copenhagen, Denmark

**Keywords:** chronic pain, hypocretins, narcolepsy, orexins, pain perception

## Abstract

Chronic pain prevalence is increased in the sleep disorder narcolepsy, but the underlying reason for this remains debated. This mini review summarizes and discusses current evidence on links between narcolepsy type 1 (NT1) and type 2 (NT2) and chronic pain, exploring whether pain is best understood as a consequence of the pathophysiology or secondary to its manifestations and comorbidities. Registry-based and self-reported data indicate a high prevalence of chronic pain in NT1, while objective pain tests show inconsistent differences between NT1 and healthy individuals. This discrepancy suggests involvement of secondary mechanisms not readily captured by experimental pain testing. Comparable pain prevalence in NT1 and NT2 also speaks against orexin deficiency as a sole driver of chronic pain in NT1. Alternatively, altered dopaminergic signaling downstream of orexin could be a shared contributor to pain in NT1 and other fatigue-related conditions. Finally, the presence of activating autoantibodies in NT1 suggests a possible immune-mediated component for some patients. Overall, chronic pain is highly prevalent in both NT1 and NT2. It likely arises from a combination of neurobiological and psychological factors, with immune mechanisms as a possible additional contributor, rather than, in NT1, orexin dysfunction alone. This perspective highlights the clinical need for more holistic treatment approaches and for future studies with improved case-control matching, larger NT1 samples, especially among patients with chronic pain, and more detailed comparisons with NT2.

## Introduction

Type 1 Narcolepsy (NT1) is a rare, multifactorial brain disorder belonging to the group of central disorders of hypersomnolence with a prevalence of 200–500 cases per million. It is caused by complex mechanisms leading to the selective loss of orexin (OX) producing neurons (Kornum et al., 2017; Liblau et al., 2024). The exact nature of the disease is debated, but studies suggest that immune processes, genetic predisposition and environmental triggers serve as crucial factors. In contrast, narcolepsy type 2 (NT2) remains poorly understood despite its large symptomatic overlap with NT1, as OX levels are typically within the normal range, and cataplexy, a hallmark symptom of NT1, is absent (Kornum et al., 2017; Bassetti et al., 2019). In addition to the characteristic pentad of narcolepsy symptoms: excessive daytime sleepiness (EDS), disrupted sleep patterns, cataplexy (only NT1), sleep paralysis and hypnagogic hallucinations, patients frequently experience a range of physical and psychiatric comorbidities. Among the most prevalent are obesity, obstructive sleep apnea, depression, cognitive and autonomous dysfunctions (Krahn et al., 2022; Jennum et al., 2013). Patients also report chronic pain syndromes such as migraines and lower back pain (Lipford et al., 2024). Despite the severe impact chronic pain can have on the quality of life of a patient, this area remains underexplored in the context of narcolepsy, and it remains debated whether there is an interplay between OXs and pain modulation. A bidirectional relationship between sleep disturbances and pain has long been recognized, with chronic pain being a shared feature in many sleep disorders (Finan et al., 2013). However, a direct role of OX deficiency also remains plausible, as the OX system modulates pain sensitivity directly or indirectly through regions involved in both sleep and nociception (Bartolucci et al., 2023).

Here, we review the literature on chronic pain in narcolepsy and consider the relative contributions of orexin deficiency and secondary factors such as hypersomnia and comorbidities. The review is based on available clinical and mechanistic literature on chronic pain in narcolepsy, following PRISMA guidelines (Page et al., 2021). The search strategy is described in the Supplementary materials, and Supplementary Table 1, and the study identification and selection can be seen in Figure 1.

**Figure 1:**
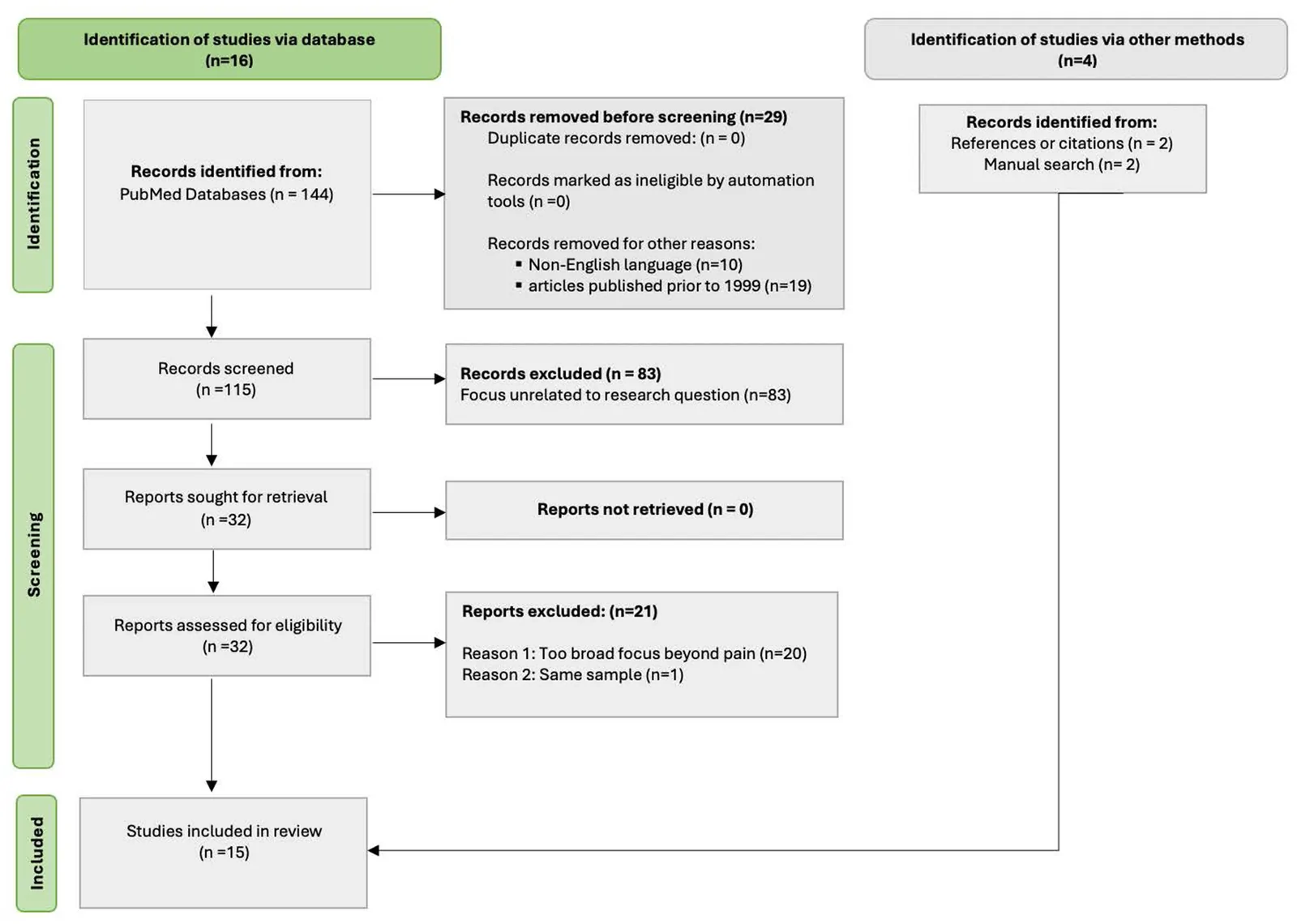
“PRISMA flow chart” illustrating the search strategy. PRISMA-style flow diagram illustrating study identification and selection. Further details of the literature search and study selection are provided in the Supplementary material (Page et al., 2021).

## Chronic pain is common in narcolepsy across study designs

High rates of chronic pain are consistently reported in narcolepsy, with prevalence estimates ranging from 32.8 to 84.84% in cross-sectional studies (Dauvilliers et al., 2011; Cremaschi et al., 2018). These estimates were based on different operational definitions: Dauvilliers et al. (2011) classified pain occurring at least monthly as frequent chronic pain, whereas Cremaschi et al. (2018) defined chronic pain as pain lasting ≥3 months. Pain definitions and assessment methods across the clinical studies are summarized in Supplementary Table 2.

This pattern of increased pain burden is further supported by observational studies (Jennum et al., 2013; Lipford et al., 2024) and one community-based cohort study (Cohen et al., 2018), all reporting increased pain-related comorbidity in narcolepsy (sample sizes 66-2057). Four studies included both NT1 and NT2 patients in their samples (Jennum et al., 2013; Lipford et al., 2024; Cremaschi et al., 2018; Cohen et al., 2018) however, two did not distinguish between the subtypes upon analysis (Jennum et al., 2013; Lipford et al., 2024). Dauvilliers et al. (2011) compared patients' self-reported levels of pain to assessments made by a close acquaintance (e.g., partner, friend, or relative) and by a physician. While 83% of acquaintances' evaluations aligned closely with patients' reports, physicians significantly underestimated pain severity in 60% of cases (*p* < 0.01), reinforcing the need for greater clinical awareness. Regarding subtype comparisons, Cremaschi et al. (2018) report chronic pain in 84.84% of NT1 patients (*n* = 33) and 75.75% of NT2 patients (*n* = 33).

## Pain in narcolepsy cannot be attributed to comorbidities alone

Another recurrent observation across studies is the high prevalence of comorbid sleep and mood disorders (e.g., insomnia, obstructive sleep apnea, anxiety, and depression) in NT1. This calls into question whether such comorbidities act as a confounding factors - an important consideration, given that mood disorders are known to influence pain perception (Scaini et al., 2025; Finan and Smith, 2013). Lipford et al. (2024) addressed this by controlling for comorbidity through propensity score matching. Beyond standard demographic characteristics (e.g., age, sex) participants were matched on total number of diagnoses, improving comparability between groups. Despite this approach, chronic pain remained significantly more prevalent in narcolepsy patients compared to the control cohort suggesting that measured comorbidities do not fully explain the association, although residual confounding cannot be excluded. Both Dauvilliers et al. (2011) and Lipford et al. (2024) also explored associations between depression, sleep disturbances, and pain. While both studies proposed bidirectional relationships, neither established causality. Nevertheless, it has been suggested that depression and sleep disturbances act synergistically to increase risk of chronic pain rather than as isolated variables. In a broader population-based study, Jiang et al. (2024) found that depression and sleep disorders each increased chronic pain risk individually, but their combined effect nearly quadrupled it (OR = 3.95). Though not NT1-specific, this amplifying effect underscores the need to consider depression as confounder when addressing chronic pain in narcolepsy.

## Experimental pain testing has not demonstrated consistent alterations in NT1

An alternative approach to studying nociception is to directly measure pain sensitivity. In individuals with narcolepsy objective threshold measurements have so far failed to demonstrate any significant alterations. Spielberger et al. (2015) hypothesized that OX deficiency in NT1 might lead to altered nociception and tested this using quantitative sensory testing (QST) (Spielberger et al., 2015). The analysis was performed on a sample size of 13 patients with NT1 and 13 age- and sex-matched controls and evaluated both mechanical, cold, warm and pressure pain thresholds. No significant differences in experimental pain thresholds were found across the tested modalities. To control for potential confounding effects, sleepiness was measured before and after testing and did not differ between patients and matched control subjects. A more recent study by Bartolucci et al. (2023) extended this work by assessing muscle pain sensitivity and temporomandibular disorders in a larger NT1 cohort (*n* = 39) and matched controls. Again, no significant differences in experimental pain sensitivity were found between NT1 patients and controls. Unlike the registry- and questionnaire-based studies (Jennum et al., 2013; Lipford et al., 2024; Cremaschi et al., 2018; Cohen et al., 2018), the NT1 patients in Bartolucci et al. (2023) did not exhibit significantly elevated scores on chronic pain questionnaires. This may reflect the study's exclusion criteria, which removed individuals with conditions known to affect pain perception. As a result, the sample might not have been fully representative of the broader NT1 population, where chronic pain is frequent. Furthermore, although these studies did not demonstrate altered experimental pain thresholds in NT1, such measures assess specific aspects of nociceptive processing and may not adequately capture the mechanisms underlying chronic pain. More studies are therefore needed before a firm conclusion can be drawn.

## Autoantibodies as a potential contributor to altered pain processing

The almost equally high frequency of chronic pain in NT1 and NT2, together with the inability to identify distinct pain characteristics between the subtypes (Dauvilliers et al., 2011) weakens the likelihood that loss of OXs alone explains chronic pain in NT1. Still, a shared pathophysiology may exist beyond the OX system. A case series by Orjatsalo et al. (2021) discovered the presence of autoantibodies in NT1 patients (*n* = 10) targeting and activating G-protein-coupled nociceptive receptors. These antibodies were detected in 90% (9/10) of NT1 patients, compared to none of the healthy controls (0/100; *p* < 0.001). The prevalence of such antibodies raises the possibility of an immune-mediated contribution to altered pain processing through autoimmune activation of G-protein coupled nociceptive receptors. This interpretation is, however, based on limited evidence and should therefore be regarded as tentative.

## Subtle orexin deficits in NT2 could underlie pain symptoms

The argument against OX-deficiency as a pain contributor often relies on NT2 patients experiencing similar pain levels despite normal cerebrospinal fluid (CSF) OX concentrations. Yet, the mere detection of OX peptides in NT2 does not necessarily confirm their proper function. A study by Sakai et al. (2019) implies that CSF measurements of OXA may overestimate *functional* OX-levels, as conventional assays fail to distinguish between intact biologically active peptides and inactive degradation products (Sakai et al., 2019). Furthermore, in some NT1 patients, initially classified as “non-deficient” due to normal CSF-OX levels, the fraction of intact OX has been found to be significantly lower than in controls (Sakai et al., 2019). This suggests a possible impairment in OX-neurotransmission despite seemingly normal overall concentrations. Similarly, in NT2, a trend toward lower levels of intact OX was observed, though this difference did not reach statistical significance (Sakai et al., 2019). Therefore, CSF-OX levels in NT2 may be overestimated, with current techniques potentially masking subtle OX-dysfunction rather than reflecting a fully intact system.

## Orexin loss could alter balance between anti- and pronociception

Current evidence on the differential roles of OXA, OXB, OX1R, and OX2R in pain modulation is based largely on preclinical evidence, and its relevance to chronic pain in NT1 remains hypothetical. In this context, most discussions of orexinergic pain mechanisms in narcolepsy have focused on OXA, whereas OXB has received less attention despite its potential relevance to pain processing. Since NT1 is characterized by a severe loss of OX-producing neurons, resulting in low levels of both OXA and OXB, it is reasonable to hypothesize that this generalized loss may disrupt a fine-tuned balance between anti- and pronociception. Considering that OX1R is predominantly activated by OXA (Sakurai, 2007) and that OX1R signaling in the PAG has been linked to analgesic effects (Kaneko and Kuwaki, 2023), its reduced availability in NT1 may weaken an important inhibitory pathway. Meanwhile, OX2R, for which OXB exhibits a 10-fold greater affinity than for OX1R (Lang et al., 2004), can mediate both pro- and antinociceptive effects depending on the circumstances and location (Bartsch et al., 2004; Stanyer et al., 2024). This includes regions such as the PAG, a key structure in descending pain modulation that receives orexinergic projections (Stanyer et al., 2024). Recent animal data from Zhu et al. (2024) suggest that OXB may exert protective antinociceptive effects in neuropathic pain via suppression of neuroinflammation and oxidative stress, whereas other findings, including those summarized by Stanyer et al. (2024) propose that OX2R activation can promote nociception under certain stress-related or inflammatory conditions.

These results underscore the importance of distinguishing between OX's dual role in acute and chronic pain, as modulation of the former is largely mediated by OXA through its interaction with OX1R, while evidence suggests that chronic pain involves more complex mechanisms including contributions from both OXA and OXB via OX2R (Yamaguchi et al., 2020). In NT1, the abnormally low levels of OXA and OXB and the few remaining OX neurons signaling to only a subset of OX1R and OX2R receptors could in theory shift the balance toward heightened pain perception. Such an imbalance could explain why NT1 patients, despite a general OX deficiency, experience such varied pain profiles. However, this interpretation is speculative and based primarily on preclinical and indirect evidence. Additional research is needed to clarify the distinct roles of OXs in pain modulation (Figure 2).

**Figure 2:**
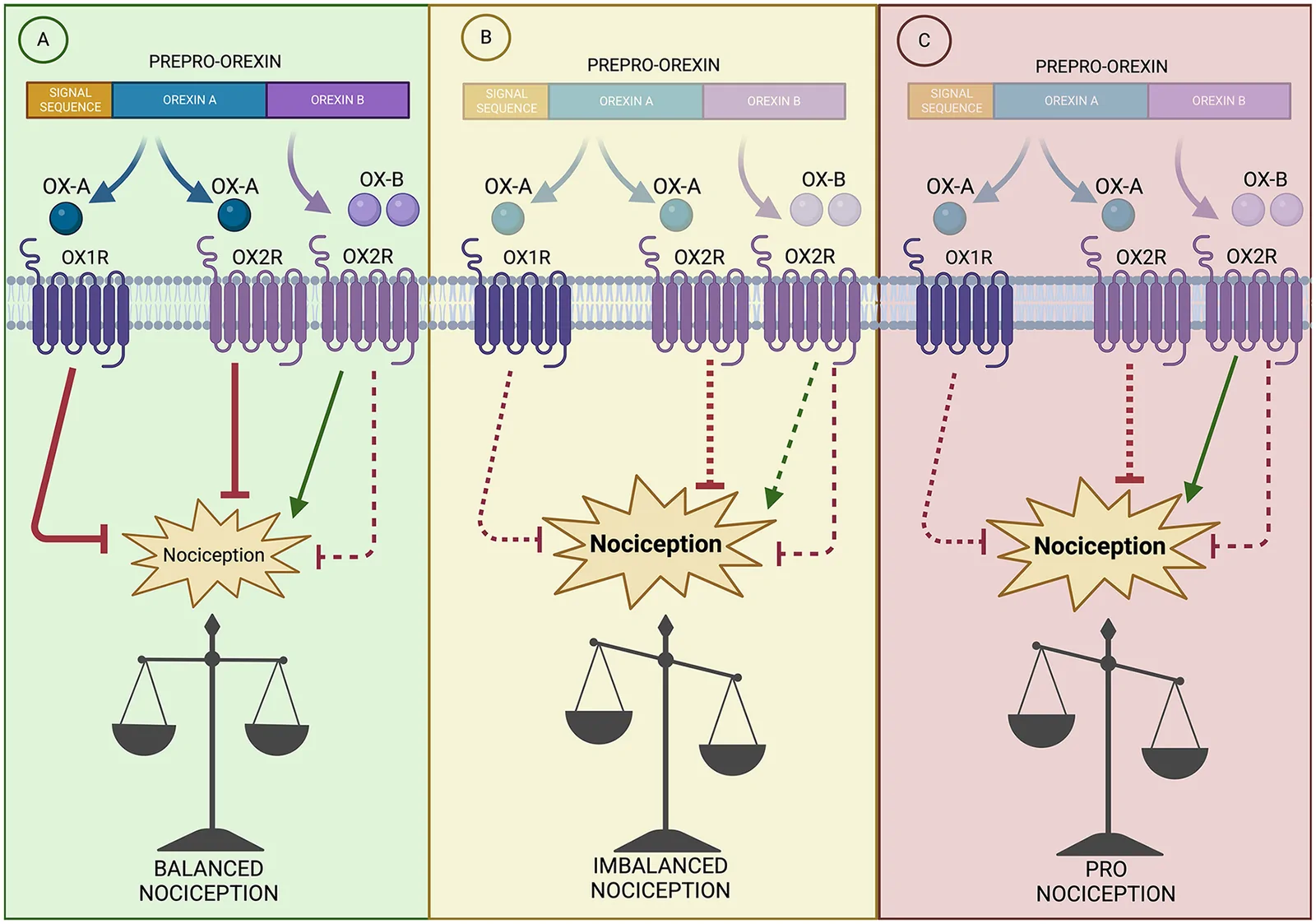
Proposed model of altered nociceptive signaling in NT1. **(A)** Experimentally supported orexinergic pathways involved in nociceptive modulation. **(B, C)** Hypothesized effects of orexin loss in NT1, in which reduced OXA/OXB signaling may disrupt the balance between anti- and pronociceptive pathways and potentially favor residual pronociceptive OXB–OX2R signaling. These mechanisms have not been directly demonstrated in patients with NT1 and are based primarily on preclinical and indirect evidence (Bartsch et al., 2004). Inspired by Stanyer et al. (2024).

## Could impaired dopaminergic tone link orexin deficiency to chronic pain?

While OXs exert direct effects through OX1R and OX2R activation, a substantial part of their modulatory function occurs indirectly through widespread projections to downstream neurons, including dopaminergic, noradrenergic, serotonergic, and opioid systems (Steiner et al., 2021). In addition to receptor activation, OX neurons co-release glutamate and dynorphin (Rosin et al., 2003; Chou et al., 2001) which further influence and complicate pain processing through excitatory and inhibitory mechanisms. Of particular interest is the dopaminergic system, which is involved in both arousal and antinociception (Finan and Smith, 2013). OX neurons innervate dopaminergic regions such as the ventral tegmental area (VTA) and promote dopamine release in the nucleus accumbens (Finan and Smith, 2013). In NT1, OX deficiency may impair dopaminergic tone, contributing not only to sleep–wake instability but potentially to altered pain processing. That said, this interpretation is based on limited clinical evidence. This hypothesis is indirectly supported by a recent case report (Barone, 2024) where a patient with narcolepsy experienced resolution of chronic migraine following treatment with armodafinil - a wake-promoting agent known to increase extracellular dopamine by inhibiting dopamine reuptake. This effect was sustained for over a year, with recurrence of pain only after discontinuation of the drug (Barone, 2024). The resolution of headache coincided with improved fatigue, suggesting a shared mechanism, possibly via dopaminergic stabilization. Although not yet confirmed in controlled studies, these observations are consistent with the hypothesis that impaired dopaminergic tone in NT1 may contribute to both pain and fatigue; this possibility warrants systematic investigation.

## Discussion

### Chronic pain in narcolepsy as a multifactorial phenomenon

A central problem in the literature is the discrepancy between the high reported prevalence of chronic pain and the absence of consistent findings in experimental pain testing. Since chronic pain is common in clinical studies, but objective pain testing remains negative, pain in narcolepsy cannot be explained by altered pain thresholds alone. At the same time, the available literature does not support the opposite conclusion either, that pain in narcolepsy is simply secondary to hypersomnia and comorbidity. Depression, sleep disturbance, fatigue, and related conditions likely contribute substantially to pain burden, but they do not capture the full picture, as studies controlling for comorbidity still found elevated pain prevalence in narcolepsy, suggesting an additional disease-related contribution.

The consistent co-occurrence of pain, fatigue, and depression in narcolepsy suggests a shared downstream mechanism; impaired dopaminergic tone secondary to orexin loss is a plausible candidate, though direct evidence remains limited.

Taken together, the literature suggests that chronic pain in narcolepsy is best conceptualized as a multifactorial phenomenon. This does not exclude a role for narcolepsy-related neurobiology, but it suggests that such contributions are likely shaped and amplified by disturbed sleep, fatigue, mood symptoms, and other comorbid factors. A more useful interpretation may therefore be to view chronic pain in narcolepsy not as only a direct consequence of disease pathophysiology or as secondary to hypersomnia and comorbidities, but as emerging from the interaction between these dimensions.

## Clinical relevance and future directions

Understanding chronic pain in NT1 and its etiology is crucial for improving diagnosis, treatment, and guiding future research. Although studies have linked NT1 to increased pain prevalence, limitations such as reliance on self-reporting and exclusion of patients with comorbidities complicate interpretation. Future studies should refine control matching and comparability by including individuals with depression, chronic pain, or insomnia but without NT, to better isolate NT1's specific contribution to altered pain processing.

A central limitation in current research on experimental pain sensitivity is the exclusion of NT1 patients with medical conditions affecting pain sensitivity. While this strengthens internal validity, it risks underrepresenting those with chronic pain. Due to the high prevalence, future research should specifically test experimental pain sensitivity in this subgroup to determine whether nociceptive processing is altered. Autoantibodies targeting nociceptive receptors, observed in NT1, could be relevant, but larger studies are needed to establish their significance. Functional assays are also needed to determine whether such antibodies have an effect on nociceptive signaling. Future studies should also clarify whether dopaminergic dysfunction secondary to OX loss contributes to pain dysregulation in narcolepsy. In parallel, the emerging concern that current CSF assays may overestimate functional OX levels (Sakai et al., 2019) underscores the need for improved diagnostic tools. Assays that differentiate intact, active OX from degraded fragments could improve accuracy, especially in NT2 and related conditions, and help identify subclinical OX dysfunction.

Clinically, reports of physicians underestimating subjective pain in NT1 patients (Dauvilliers et al., 2011) reinforce the need for greater awareness. While insight into OX and the molecular mechanisms underlying pain in NT1 are essential for developing targeted pharmacological therapies, treatment strategies should not rely solely on this approach. Because of the synergistic effect of depression and sleep disturbances on chronic pain (Jiang et al., 2024) these comorbidities must be addressed in parallel. If left untreated, their amplifying effect on pain could act as a barrier to successful treatment. This highlights the need for a holistic treatment approach, integrating pharmacological, psychological, and sleep-focused interventions to optimize outcomes in narcolepsy-associated chronic pain. Because of the distinct roles of OX1R and OX2R in pain modulation (Stanyer et al., 2024) the therapeutic potential of OX receptor subtype selective drugs should be further explored.

## Conclusion

This mini review aimed to examine the prevalence and characteristics of chronic pain in narcolepsy, explore potential neurobiological and psychological contributors, and identify gaps in the existing literature. Based on several studies it is clear that chronic pain is highly prevalent in both NT1 and NT2. Elevated depression scores were also consistent across almost all included studies, supporting the influence of secondary contributors. The possibility of an intrinsic component cannot be ruled out as available data point to OXs exerting both direct and indirect effects on pain modulation. However, current findings are insufficient to confirm a causal role. The high prevalence of chronic pain in NT2, whose pathophysiologic similarity to NT1 remains debated, complicates the question and challenges OX deficiency as the sole driver. Speculative hypotheses point to dopaminergic dysregulation and possible immune-related mechanisms as additional factors. Overall, chronic pain in narcolepsy likely arises from a multifactorial interplay between neurobiological and psychological factors, with immune mechanisms representing a possible additional contributor. Clinically, this underscores the importance of a holistic approach, addressing comorbidities such as depression, sleep disturbances, and fatigue in parallel with pain.
