# Supplementary material for: Chronic pain in narcolepsy reflects interacting neurobiological and psychological mechanisms: a mini-review
Source: Front Sleep. 2026 Sep 25;5:1930803. doi: 10.3389/frsle.2026.1930803 (PMC13650916; doi:10.3389/frsle.2026.1930803)
Supplement: Supplementary file 1 [file Supplementary_file_1.pdf]

METHODS

This manuscript was conducted based on available clinical and mechanistic literature on chronic pain in narcolepsy. Study identification and selection were documented using a PRISMA-style flow diagram. As the main purpose was to assess potential explanations for chronic pain in narcolepsy, PubMed was selected as the database of choice for material retrieval due to its extensive coverage of biomedical research.

2.1 Search Strategy and Selection

The platform was searched for publications covering the matter of interest using Medical Subject Headings (MeSH), subheadings along with free-text keywords. Two separate thematic 'blocks' were created: one for 'NARCOLEPSY' and one for 'PAIN'. To secure a wide range of aspects related to the research question and to include newer, non-MeSH indexed material, free text terms were combined within each block as shown in table 1. At last, to construct the final search string, the two blocks were combined yielding 144 publications. Before further screening, 115 articles were excluded based on the following criteria:

Inclusion and exclusion criteria

Studies were included if they:

(1) investigated chronic pain, pain perception, or nociceptive mechanisms in individuals with narcolepsy type 1 or type 2.

(2) were original research articles, including observational studies, experimental studies, and case reports.

(3) were published in English.

(4) were published between January 1999 and May 13, 2025.

Studies were excluded if they:

(1) were published prior to 1999, as the association between orexin deficiency and narcolepsy was first described in that year.

(2) did not address pain-related outcomes.

(3) focused exclusively on health-related quality of life without specific assessment of pain;

(4) were non-English publications.

To enhance scientific validity original research was prioritized. However, relevant secondary literature (e.g. reviews, commentaries) contributing important perspectives were also evaluated, although not included as part of the formal study selection.

Selection process consisted of two phases: abstract screening and full-text assessment. The initial screening aimed to identify articles that mentioned the prevalence of pain or nociceptive mechanisms in the context of narcolepsy. Although the paper's primary focus was on NT1, relevant data on patients with NT2 were considered when available to determine the potential role of OX-insufficiency in pain conditions. 32 articles met the criteria and were sought for retrieval. In the final selection phase, full-text articles were evaluated for their relevance to the research question. *Health-Related Quality of Life*-studies were excluded due to their expansive scope beyond pain and its direct comorbidities. Ultimately, eleven articles from the primary search were included. One research article, not listed in PubMed, was identified via citation-tracking and included for its novel contribution to understanding pain perception in NT1 patients. Another study was found from the reference list of an included review. In addition to the studies identified through the structured search, relevant reviews and primary literature not captured in the initial screening were included to provide biological context and support key hypotheses discussed in this manuscript. The search was conducted on May 13, 2025 by Maria Munch-Menzer.

Supplementary Table 1: *PubMed Search Protocol*

	Concept 1	Concept 2
**Key concept**	Narcolepsy	Pain
**MeSH terms and Subheadings**	"Narcolepsy"<br>"Narcolepsy/complications"<br>"Narcolepsy/ethiology"<br>"Narcolepsy/physiopathology"	"Pain"<br>"Pain/etiology"<br>"Pain/ physiopathology<br>"Nociception"<br>"Chronic pain"
**Free Text terms**	"Narcoleps*"<br>"NT1"	"Pain"<br>"Nocicept*"<br>"Pain sensivity"<br>"Pain perception"<br>"Hyperalgesia"<br>"Pain modulation"
**Hits (n=)**	7,202	1,051,498

See figure 1: 'PRISMA Flow Chart' for the complete search strategy.

Supplementary Table 2: *Pain Definitions and Assessment in Clinical Studies*

Study	Identification	Sample	Population	Controls	Study Design	Pain Definition/ Operationalization	Method	Key Findings
Dauvilliers et al (2011)	Primary search	(n=67)	NT1 (n=67)	(n=67)	Cross-sectional case-control	Self-reported pain occurring at least monthly	Standardized clinical interviews<br><br>Self-report questionnaires: MOS, FOSQ, SF-36, BDI, FSS)	1. Chronic pain significantly more common in NT1<br>2. Significantly underestimation of pain prevalence and impact from physicians<br>3. Mood disorders and sleep quantity as significant determinants for pain
Cremaschi et al (2018)	Primary search	(n=66)	NT1 (n=33)<br>NT2 (n=33)	(n=66)	Cross-sectional case-control	Chronic pain defined as pain lasting ≥3 months<br><br>Pain intensity assessed using NRS and pain characteristics using MPQ.	Self-report questionnaires including NRS, MPQ, BDI and Pain Rating Index; semantic analysis of pain descriptors	1. High prevalence of chronic pain in NT1 (84.8%) and NT2 (75.7%).<br>2. Depression and obesity may contribute to pain.<br>3. No significant differences in pain characteristics between NT1 and NT2.<br>4. Affective and sensory pain descriptors were frequently reported.<br>5. Similar pain profiles across NT1 and NT2.
Jennum et al. (2013)	Primary search	(n=757)	NT1 & NT2 (n=757)	(n=3013)	Retro- and prospective observational cohort study Case-control	Pain-related comorbidity identified from registry diagnoses.	Health registry data	1. Chronic pain and comorbidities elevated before and after diagnosis; suggests early or intrinsic involvement.<br>2. Mortality not significantly elevated
Cohen et al. (2018)	Primary search	(n=68)	NT1 (n=28)<br><br>NT2 (n=40)	(n=272)	Longitudinal, prospective community-based cohort study Case-control	Chronic low back pain identified from medical records.	Patient records<br><br>Interviews	High prevalence of chronic pain and comorbidities both at time of diagnosis and at follow up

Lipford et al (2024)	Primary search	(n=2057)	NT1 + NT2 (n=2057)	(n=2057)	Observational, retrospective<br><br>Propensity-matched case-control	Chronic pain syndrome identified from electronic health record diagnoses.	Electric health record-based comorbidity analysis	1. Higher chronic pain prevalence in NT1 despite propensity matching; points to independent association.<br>2. Chronic pain syndrome more than twice as likely compared to controls (OR=2.3, p<0.001).<br>3. Acknowledges the potential influence of comorbid conditions such as depression
Spielberger et al. (2015)	Primary search	(n=13)	NT1 (n=13)	(n=13)	Case-control	Experimental pain sensitivity assessed by standardized QST.	Quantitative Sensory Testing<br><br>Self-report questionnaires: (SSS, ESS)	No significant difference in pain perception vs. controls regardless of stimuli
Bartolucci et al. (2023)	Citation tracking	(n=39)	NT1 (n=39)	(n=39)	Cross-sectional case-control	Pain sensitivity assessed by PPT and pain-related questionnaires.	Pressure Pain Thresholds (PPT)<br><br>Self-report questionnaires: (PHQ-9, PHQ-15, GCPS 2.0, JFLS-20, GAD-7, OBC)	1. No significant difference in pain threshold of central sensitization evident<br>2. Significantly higher depression rates (p<0.05), possibly contributing to symptom magnitude.

BDI = Beck Depression Inventory, ESS = Epworth Sleep Scale, FOSQ = Functional Outcomes of Sleep Questionnaire, FSS = Fatigue Severity Scale, GAD-7 = Generalized Anxiety Disorder, GCPS = Graded Chronic Pain Scale, JFLS = Jaw Functional Limitation Scale, MOS = Medical Outcomes Study, MPQ = McGill Pain Questionnaire, NRS = Numeric Rating Scale , OBC = Oral Behavior Checklist, PHQ = Patient Health Questionnaire, SSS = Stanford Sleepiness Scale, SF-36 = Short-Form 36-item QoL
